# Rapid Prototyping for *In Vitro* Knee Rig Investigations of Prosthetized Knee Biomechanics: Comparison with Cobalt-Chromium Alloy Implant Material

**DOI:** 10.1155/2015/185142

**Published:** 2015-03-24

**Authors:** Christian Schröder, Arnd Steinbrück, Tatjana Müller, Matthias Woiczinski, Yan Chevalier, Patrick Weber, Peter E. Müller, Volkmar Jansson

**Affiliations:** Department of Orthopaedic Surgery, Physical Medicine and Rehabilitation, University Hospital of Munich (LMU), Campus Grosshadern, Marchioninistraße 15, 81377 Munich, Germany

## Abstract

Retropatellar complications after total knee arthroplasty (TKA) such as anterior knee pain and subluxations might be related to altered patellofemoral biomechanics, in particular to trochlear design and femorotibial joint positioning. A method was developed to test femorotibial and patellofemoral joint modifications separately with 3D-rapid prototyped components for *in vitro* tests, but material differences may further influence results. This pilot study aims at validating the use of prostheses made of photopolymerized rapid prototype material (RPM) by measuring the sliding friction with a ring-on-disc setup as well as knee kinematics and retropatellar pressure on a knee rig. Cobalt-chromium alloy (standard prosthesis material, SPM) prostheses served as validation standard. Friction coefficients between these materials and polytetrafluoroethylene (PTFE) were additionally tested as this latter material is commonly used to protect pressure sensors in experiments. No statistical differences were found between friction coefficients of both materials to PTFE. UHMWPE shows higher friction coefficient at low axial loads for RPM, a difference that disappears at higher load. No measurable statistical differences were found in knee kinematics and retropatellar pressure distribution. This suggests that using polymer prototypes may be a valid alternative to original components for *in vitro* TKA studies and future investigations on knee biomechanics.

## 1. Introduction

Nearly one-fifth of the patients are unsatisfied after primary total knee arthroplasty (TKA) [1]. An extensive part of patients complain about pain in the anterior knee joint, which should be associated, amongst other things, with an increased retropatellar pressure [2–4]. Instabilities, subrespectively, luxation, and fractures of the patella after implantation are further complications and reasons for revision [5–9]. These given facts show precisely the requirement of research and development on this part of the knee joint.

It is common knowledge that knee biomechanics after total knee arthroplasty (TKA) are altered due to changes in pressure distribution as well as the kinematics pattern [3, 10, 11]. Workgroups showed that an external or internal rotation of the femur component changed the retropatellar pressure and kinematics [12–14]. However, these workgroups use commercial prostheses and therefore both the alignment of the trochlear groove and the flexion gap changed while rotating the femur component. The tendon stresses of the collateral and posterior cruciate ligament as well as the knee flexion axis were modified by using this method. Specially designed prototypes have been proposed to analyze the influence of different trochlea alignments as well as different trochlear shapes for total knee arthroplasty without influencing the femorotibial positioning of the prosthesis [15].

Additive technology, or rapid prototyping, is a current trend in the industry to produce prototypes in the early stage of development allowing evaluating the effects of design. These procedures are used in medicine amongst others for the reconstruction of jaw and face bones [16, 17], but also in the area of tissue engineering to produce scaffold for the cell population [18]. Patient-specific cutting guides made by rapid prototyping are newly offered to the orthopedic field to specify the implantation of prostheses, in particular for knee surgery [19, 20]. The manufacturing of patient-specific knee prostheses made by rapid prototyping for scientific research is still in the early stage. Prototypes made of metal—for example, manufactured by laser sintering—are still expensive at this moment and require additional treatment, such as polishing to achieve surface roughness representative of the commercial prosthesis. Alternatively, methods such as 3D-printing or molding can be used to create polymer prototypes. In particular, 3D-printing is a quick and inexpensive method which allows high geometrical accuracy. However, the use of polymers rather than metallic alloys results in prototypes of reduced mechanical properties. It is unknown if such materials may be suitable for* in vitro* testing, in particular for* in vitro* knee kinematic studies which typically subject the knee joint to reduced body weights for a limited number of loading cycles.

This study therefore sought to determine if the use of rapid prototyped prostheses can serve as an alternative to the standard prostheses for* in vitro* studies for knee, in particular for the study of retropatellar biomechanics.

To test this issue, the sliding friction parameters of the standard bearing combination in TKA (ultrahigh molecular weight polyethylene versus cobalt-chromium alloy) were firstly measured and compared to the rapid prototype material. Additionally, PTFE was used as a counterpart against both femoral component materials in the friction test, because it is often used to protect the pressure sensitive foil against shear. The foil is sutured on the patella surface and has therefore contact with the femoral component [3, 11, 21].

Then, in a second step, rapid prototyped femoral components of a commercial prosthesis design were created and implanted in seven cadaver knee specimens. These were tested in a custom-made dynamic knee rig under standardized conditions while recording retropatellar pressure and knee kinematics, and these measurements were compared with the ones obtained for identical tests for standard components made of cobalt chromium in the same knee specimens.

## 2. Materials and Methods

### 2.1. Prostheses and Test Samples

**Figure 1:**
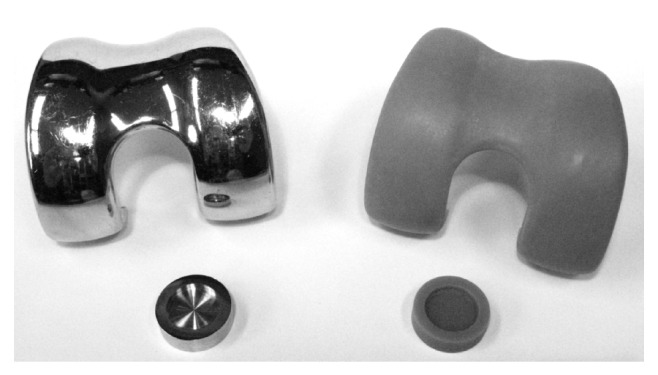
The Columbus CR prosthesis (Aesculap AG, Tuttlingen, Germany; top) and the test pieces (bottom) for the friction test made of the standard material (CoCr29Mo6) on the left and the rapid prototype material (RGD840) on the right side.

Femoral components of a fixed bearing knee prosthesis (Columbus CR, Aesculap, Tuttlingen, Germany) manufactured from casted CoCr27Mo6 (standard prostheses material; SPM) and rapid prototype material (RPM; RGD 840 vero blue, Stratasys GmbH, Frankfurt) were obtained from the manufacturer (Figure 1). The material specifications of the RPM are shown in Table 1. Original CAD data were used to produce rapid prototypes for the femoral components in sizes 2 until 5 for each side (left/right knee) with a professional 3D-printer (Object Eden 350, Rehovot, Israel). These specimens were printed in thin layers (down to approximately 50 *μ*m) of a liquid photopolymer resin (Table 1), which immediately polymerized under UV-light. Afterwards the rapid prototyped prostheses were polished submerged with fine grained sandpaper (up to grain size 1000). Finally, a roughness of* R*_*a*_ = 0.44 *μ*m was reached for the surrogates, while the prostheses made of SPM reached* R*_*a*_ < 0.05 *μ*m.

Tibial insert was made of UHMWPE (GUR 1050).

For the ring-on-disc friction test ring-shaped samples were produced of SPM and RPM. The discs which were used as counterparts in the test setup were made of ultrahigh molecular weight polyethylene (UHMWPE) and polytetrafluoroethylene (PTFE; HighTechflon, Konstanz, Germany).

### 2.2. Ring-on-Disc Friction Test

#### 2.2.1. Ring-on-Disc Rig

**Figure 2:**
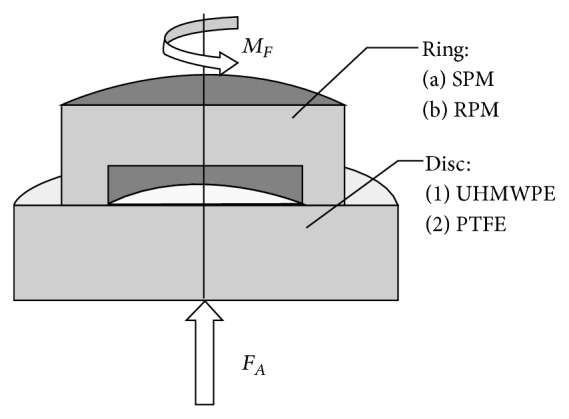
Schematic setting of the ring-on-disc rig with the demonstration of the axial compression force (*F*_*A*_) and the friction torque (*M*_*F*_) as well as the tested material combinations. The rotation of the ring occurs about the rotation axis, which is identic to the axial load transmission.

A tribological test setup according to ISO 6474 and Huber et al. was used to measure the friction coefficient (*μ*_*R*_) [22]. The ring (friction area 160.2 mm²; radius_external_ = 10 mm; radius_internal_ = 7 mm) rotated periodically with 1 Hz on the disc (diameter = 25 mm) with an amplitude of ±25°. Axial compression between ring and disc was adjusted with a manually controlled trapezoidal spindle. The compression was measured with a force transducer (HBM, Darmstadt, Germany), while the friction moment was detected with a beam using a half bridge strain gauge (HBM, Darmstadt Germany). This moment was converted into a force by using the geometrical parameters of the specimen (Figure 2). Both sensors were connected to a personal computer using an analog digital converter (compactDAQ with NI 9237 & NI 9236 modules; National Instruments, Austin, USA) and a self-written program code on LabVIEW (Version 2011, National Instruments, Austin, USA) to record sensors data continuously with a sample rate of 1000 samples per second.

#### 2.2.2. Friction Test

Forty-eight hours before testing, all specimens were conditioned in a synovial replacement composed of newborn calf serum (S0125; Biochrom AG; Berlin, Germany) diluted with deionized water to reach a protein concentration of 30 g/L. Lubrication was secured using the same fluid during the test. The sliding friction coefficient (friction coefficient) of six specimens per material combination (Table 2) was measured in 250 N steps beginning from 500 N to 1500 N axial compression under ambient temperature. Friction force was measured five times at each axial load step. The friction coefficient was calculated by dividing the friction force by the axial force.

#### 2.2.3. Statistics

An unpaired 2-way ANOVA (material pairing and axial load) with a Tukey post hoc test was used to compare each group (SPSS 21, IBM). Significance was approved with *P* < 0.05. Additionally the mean difference (MD) and the confidence intervals (CI) were presented.

### 2.3. Knee Rig Investigation

#### 2.3.1. Knee Rig

**Figure 3:**
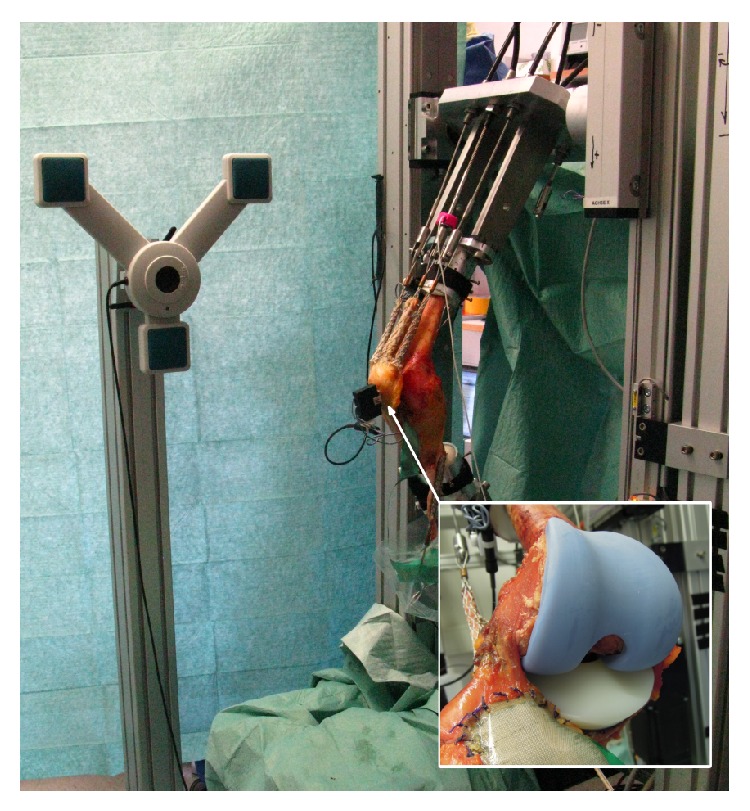
Knee rig with a mounted specimen with implanted TKA; knee kinematics were measured with an ultrasound markers on femur, tibia, and patella; by removing the anterior cables, pressure sensitive foil covered with Teflon tape behind the patella and prosthesis were visible (magnification-box).

The custom knee rig (Figure 3) simulates a loaded squat with a human specimen using two linear drives as presented in a previous publication [11]. The first actuator flexes and extends the knee with a constant velocity. Two angle sensors (8820 Burster, Gernsbach, Germany) in the upper “hip” and lower “ankle” joint are used to measure the flexion angle of the knee joint. The second stepper drive simulates the quadriceps muscle. The resulting force is measured with miniature force transducers (8417-6002 Burster, Gernsbach, Germany) near the tendons. Additionally hamstrings, vastus lateralis, and medialis muscle are simulated with 2 kg masses. A six degree of freedom force-moment-sensor measures the ground reaction (FN 7325-31 FGP Sensors, Cedex, France).

All sensors are amplified up to maximum 10 volts and digitalized with an 18-bit analog digital converter (PCI 6281 National Instruments, Texas, USA). The rig is controlled with a quad-core personal computer. Four parallel real-time program loops (LabVIEW, Austin, US) for each core are necessary to control the knee rig, while the first loop acquires the sensor data. The second loop is a parabolic PID-force-control loop to hold the ground reaction force constant with the loaded quadriceps muscle. The third loop controls the flexion and extension of the knee in a constant velocity. The last loop writes the sensor data into an ASCII-File.

For this study all specimens were tested with a ground reaction force of 50 N, a velocity of 3°/s, and a squat from 20° to 120° flexion and extension back to 20°.

Retropatellar pressure distribution (Tekscan, Boston, US) and femorotibial (ap-translation, femorotibial rotation) and patellofemoral (tilt, rotation, shift defined in [23]) kinematics were acquired during the squat with an ultrasound three-dimensional motion analysis system (CMS 20 Zebris, Isny, Germany) ensuring an accuracy of 0.1 mm and 0.1°.

#### 2.3.2. Cadaver Preparation and TKA Implantation

Seven human knees (57.7 ± 13.0 years; 177.7 ± 6.3 cm; 82.4 ±11.9 kg; 5 males, 2 females) were tested under weight-bearing conditions. The specimens were standardized in length with a femur cut 20 cm proximally and a tibia cut 15 cm distally from the epicondyle axis, respectively. The fibula head was fixed on the tibia using a cortical screw. Muscle and fat tissues were carefully removed from the tendons and bones. Finger traps were connected to the tendons and suture wires (Fibrewire, Arthrex, Karlsfeld, Germany) were used to fix the tendon into the metallic mesh of the traps. The diaphyses of the femur and tibia were embedded into pots with epoxy resin which were then mounted on the knee rig.

A pressure sensor (K4000; Tekscan; Boston, US), protected with an additional thin layer of PTFE-tape (Thickness: 125 *μ*m, HighTechflon, Konstanz, Germany), was fixed at the articulating surface of the patella with sutures (Novosyn, B. Braun, Melsungen, Germany), with the osteophyte of the patella previously removed to ensure better contact. Before, the sensor was conditioned for 20 loading cycles and calibrated with a 2-point calibration line computed by the measurement software (I-Scan 6.1). The axial pressure for calibration and conditioning was applied with a material testing machine (Zwick Z010, Ulm, Germany).

Additionally a tripod of three ultrasound markers was attached to each bone and anatomic landmarks were identified on the specimen to define kinematic pattern in relation to the coordinate system of each bone.

An experienced surgeon (A. S.) implanted the prostheses as indicated by the manufacturer using a subvastus approach in the tibia first technique. The tibia was resected perpendicular to an intramedullary rod, while a slope of 3° was included in the inlay. A gauge instrument was used to balance flexion and extension gap. Then, an intramedullary rod was used to align the femoral component in a 4°–6° valgus rotation relative to the bone axis of the femur. The femural component was then rotated parallel to the anatomical transepicondylar axis, priorly defined using an inserted K-wire.

After implantation the specimens were tested in the knee rig by acquiring pressure and knee kinematics data without additional lubrication. Randomized change between the SPM and the RPM prostheses for each specimen allowed testing the implants consecutively. Between each test cycle the biological materials were prevented for dehydration by putting saline-soaked scarfs on the surface of the specimen.

#### 2.3.3. Statistics

Results for the RPM were plotted against the SPM results at 5° intervals of flexion angle. A Deming-regression was performed for each specimen and each parameter to calculate the slope of the regression line. Equality criterion implies a linear regression whose slope is not significantly different to 1 using a one sample* t*-test.

## 3. Results

### 3.1. Coefficient of Sliding Friction of the Ring-on-Disc Test

Both material combination and axial load influence the sliding friction coefficient in the ring-on-disc rig (*P* < 0.001).

**Figure 4:**
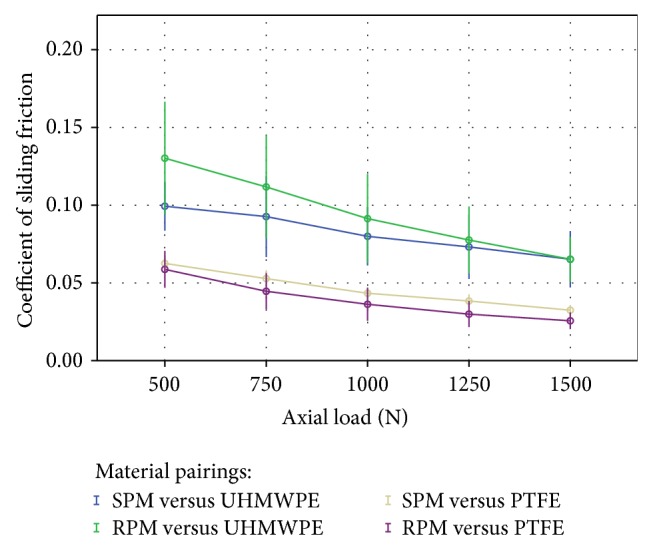
Dependence of the coefficient of sliding friction with rising axial load for the tested parameters; whiskers define the 95% confidence interval.

With increasing load, a decrease of the friction coefficient was measured in the four groups (*P* < 0.001). The friction test with the bearing partner UHMWPE provided significant higher friction coefficients than with PTFE (*P* < 0.001; Figure 4). There was no interaction between load and material combination measurable (*P* = 0.52).

The material combinations SPM and RPM against PTFE showed no differences (MD: 0.0068 (CI: −0.0047; 0.018)) in the friction coefficient (*P* = 0.413). In contrast, there was a significant difference (MD: −0.013 (CI: −0.025; −0.0016)) between the material combinations SPM, respectively, and RPM to UHMWPE (*P* = 0.013). Pairwise comparison revealed that the difference between SPM and RPM at 500 N (3.1 MPa) is significantly increased (MD: −0.031 (CI: −0.051; −0.011) *P* = 0.002). That difference tends to disappear with increasing axial loads (*F*_*A*_ = 750 N MD: −0.019 (CI: −0.039; 0.001) *P* = 0.06; *F*_*A*_ = 1000 N MD: −0.011 (CI: −0.031; 0.008) *P* = 0.3;* F*_*A*_ = 1500 N MD: 1.5·10^−5^ (CI: −0.020; 0.020) *P* = 1.0; Table 3).

### 3.2. Knee Rig Study

**Figure 5:**
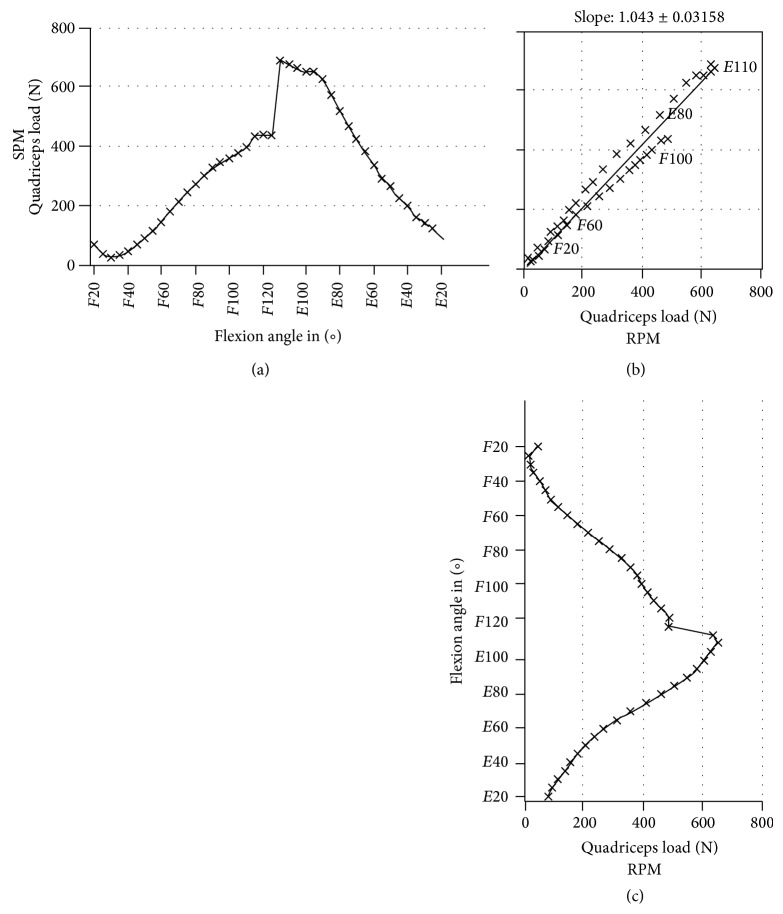
The left plot shows the quadriceps load in N against the flexion angle of the SPM and the plot at the bottom shows the flexion angle against the quadriceps load of the RPM. The diagram in the center is a projection of both plots and compares the RPM material (*y*-axis) and the SPM (*x*-axis). Angles are identified in the upper right corner plot with flexion (*F*), extension (*E*) angle. The Deming-regression line with slope and standard deviation of the slope (crosses) are shown.

For each measured specimen and each parameter, a linear regression was calculated by Deming-regression.  Figure 5 represents a typical result obtained for quadriceps load measured for one specimen with both types of implant materials.

**Figure 6:**
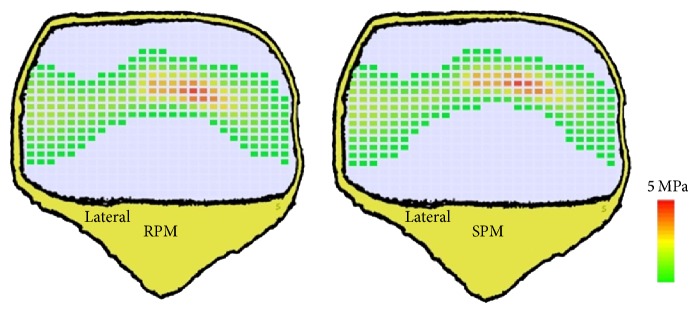
Retropatellar pressure distribution of both prosthesis materials (left: rapid prototype material; right: standard prosthesis material) at a flexion angle of 120° in one representative specimen.

No significant differences were measured in the knee rig between the two tested materials (Table 4). A maximum quadriceps load of 647 ± 131 N with RPM and 620 ± 88 N with SPM was necessary to extend the cadaver knee and caused comparable results during the whole cycle (*P* = 0.69). Furthermore the maximum retropatellar peak pressure of 6.92 ± 2.01 MPa with RPM and 6.83 ± 1.79 MPa with SPM showed no significant differences (*P* = 0.53). Little deviations could be noticed at the maximum of the contact area (Figure 6). RPM showed a tendency to a higher contact area with 402 ± 63 mm² compared to the SPM with a maximum contact area of 380 ± 61 mm². However, the change in contact area was not significant (*P* = 0.31). Similar behaviors between the RPM and the SPM in terms of kinematics of the femorotibial joint were depicted. Equivalent results of the patellar tilt of 4.95° ± 1.93° with RPM and 4.87° ± 1.86° with SPM as well as the patellar rotation of 5.29° ± 3.72° with RPM and 5.13° ± 3.47° with SPM could be obtained. The measurement of the lateral shift of the patella provided an average value of 4.87 mm (SPM ±2.0 mm; RPM ±2.2 mm) for both materials.

## 4. Discussion

Prototype constructions used in cadaver studies provide a useful way to investigate the biomechanical interaction between the patellofemoral and tibiofemoral joints separately after TKA. For research purposes Walker et al. produced several types of polymer prostheses by stereolithography [24]. Any validation of these prototypes and the proof of a similar biomechanical behavior compared to original prosthesis were not included in these studies. However, so far little is known about differences that may occur between standard implant materials and rapid prototype polymers in biomechanical studies at the knee joint. Our study is the first to demonstrate that prostheses made from RPM behave comparably to original prostheses in the knee rig.

Our study goal was to evaluate the usability of polymer rapid prototypes compared to the standard cobalt-chromium alloy prostheses for patellofemoral joint research. We expected first that the friction coefficients of both standard prosthesis material and rapid prototype material in different combinations of counterface material coupling were not different. Second, we expected that, for the weight-bearing loads typically involved in* in vitro* kinematic testing of the knee, the rapid prototyped materials may provide result of motion patterns and patella-contact forces, which are not significantly different from the standard clinical materials.

Our study results do not validate our first hypothesis. Whereas significant differences of the friction coefficient of the patellofemoral material combinations (PTFE) were not found, there were significant differences of SPM and RPM combinations with UHMWPE as counterface at a low axial load of 500 N (*P* = 0.031). With increased axial pressure, however, the friction coefficient of both materials against UHMWPE converged until no difference was measurable at 1500 N (*P* = 1). This difference might be a result of the higher surface roughness of the surrogates compared to original one.

Our second hypothesis was validated by tests in the knee rig. Indeed, comparable behaviors of SPM and RPM for the analyzed parameters were observed. Based on this, we believe that the tested RPM may be suitable for the* in vitro* testing of prostheses and the kinematical analyses in the knee rig, despite friction differences with standard prosthesis materials at low loads, when used in combination with UHMWPE.

A few limitations must be accounted in analyzing our results which may affect the validity of our conclusions to other study contexts. First, the measured coefficient of friction of material combinations depends on an amount of parameters: surface roughness, the used lubrication, the velocity, and the used axial load [25–29]. Small differences can also be obtained with the use of different friction rigs. In this study a rebuilt ring-on-disc simulator according to ISO 6474 was used, which simulated a radial sliding of a ring on a disc. On the other hand, studies such as the ones from Saikko were based on a pin-on-disc-test rig, while Wang et al. used a rig where the coefficient of friction was measured in a total hip prosthesis.

For each material combination a consistent reduction of the coefficient of friction was documented while increasing axial load. This obtained behavior was supported by Saikko and Wang et al. [30, 31]. Both of them determined an exponential decrease of the sliding friction coefficient of UHMWPE compared to cobalt-chrome alloy by increasing loads (Table 5).

To avoid shear forces in the sensitive pressure film, many workgroups covered the film with PTFE-tape [3, 11, 21]. Therefore the cartilage of the patella was replaced by a PTFE foil which has a friction coefficient ranging between 0.03 and 0.06 against SPM. Friction measurements on hip hemiprostheses showed similar friction coefficients between 0.02 and 0.11 for cartilage against surgical steel [32] and 0.02 up to 0.2 and 0.02 to 0.38 compared to cobalt-chrome alloy [33, 34]. However, such differences may not affect our conclusions in the current study design, as both prosthesis materials were subjected to similar testing conditions.

A second limitation comes from testing conditions in the knee rig. A small ground load was used in the knee rig, which may be a limitation for extrapolating our results to studies where higher loads would be simulated. However, Müller et al. and Victor et al. showed that reduced loads resulted in an amplitude change but do not alter the progression of the measured data [35, 36]. The simplified testing conditions and lower loading magnitudes therefore reduce the material requirements needed for the* in vitro* testing of knee prostheses. Biomechanical aspects such as wear, mechanical, and fatigue resistance as well as biocompatibility are minorly relevant for kinematic tests with human cadaver knees. However, differences in friction behavior can alter kinematics and contact behavior in the specimen. Therefore it is important that the results of the knee rig study affirm both, the same intra-articular pressure and knee kinematics by the use of rapid prototyped and standard prostheses material.

A third limitation is that both RPM and SPM materials were compared in only seven knee specimens. From statistical point of view, the number of specimens tested might not fully exclude a type II error. Thereby, the number of specimens is comparable to other studies [11, 14, 36–39].

The usage of a single knee prosthesis design with a rather flat-shaped trochlea can also be a forth limitation in our study. Therefore the generalized transfer of these results to other knee replacement systems is not possible. Moreover only the typical material of the described 3D-printer could be analyzed, so the transferability to other printable materials and surface roughness is restricted. Despite these limitations, we believe that our pilot study successfully demonstrated the validity of using rapid prototype implants made of polymers as surrogates in kinematic testing of the knee. There are many benefits to using prototyped knee prosthesis for* in vitro* studies. Firstly, a separately biomechanical examination of the femorotibial and patellofemoral joint is possible. Secondly, different prostheses can be implanted on the same bone cuts, which enable quick and accurate change of the component without additional bone resections. Thirdly, different prosthesis alignments are feasible with the use of the same bone cuts, by modification of the articulating surface geometry only. Fourthly, a test of different prototypes in the same specimen is possible without reoperation and therefore, due to the paired observations, fewer specimens may be necessary to reach the same statistical outcome.

## 5. Conclusion

This pilot study showed that knee prostheses produced from rapid prototype polymer can produce knee kinematics and retropatellar pressure distributions comparable to those obtained with original knee prostheses in the knee rig. Therefore it is possible to use this technique to get more comprehension of TKA biomechanics. Furthermore, consecutive biomechanical analyses of various knee prostheses designs could be analyzed in the same knee without any reoperation of the joint* in vitro*. Future studies will focus on modifying implant orientations for better understanding of the effects of malignment on patellofemoral joint mechanics and the relation to pain experienced by patients.

## Figures and Tables

**Table 1:** Material properties of the photopolymer resin.

Parameter	RGD 840 vero blue
Ingredients	Several acrylate oligomers; acrylic monomer; isobornyl acrylate; photoinitiator
Modulus of elasticity	2000–3000 MPa
Tensile strength	50–60 MPa
Elongation to break	15–25%
Shore hardness	83–86 (Scale D)
Rockwell hardness	73–76 (Scale M)

**Table 2:** Number of tested specimens subdivided in the four material combinations; UHMWPE and cobalt-chromium alloy are the common bearing material for knee arthroplasties; PTFE is not used *in vivo* but serves protection of the retropatellar pressure sensitive foil during *in vitro* examinations.

Femoral prosthesis material	CoCr27Mo6 (SPM)	RGD 840 vero blue (RPM)
UHMWPE (femorotibial counterpart)	*n* = 6	*n* = 6
PTFE (patellofemoral counterpart)	*n* = 6	*n* = 6

**Table 3:** Descriptive statistics of the friction coefficients with standard deviation of the tested material combinations at different axial loads (axial pressure).

Axial load (pressure)	SPM versus UHMWPE	RPM versus UHMWPE	SPM versus PTFE	RPM versus PTFE
500 N (3.1 MPa)	0.099 ± 0.015	0.130 ± 0.035	0.063 ± 0.005	0.059 ± 0.011
750 N (4.7 MPa)	0.093 ± 0.025	0.111 ± 0.034	0.053 ± 0.005	0.044 ± 0.011
1000 N (6.2 MPa)	0.080 ± 0.018	0.091 ± 0.029	0.043 ± 0.004	0.036 ± 0.010
1250 N (7.8 MPa)	0.073 ± 0.020	0.078 ± 0.025	0.038 ± 0.004	0.030 ± 0.008
1500 N (9.4 MPa)	0.065 ± 0.017	0.065 ± 0.021	0.032 ± 0.003	0.026 ± 0.005

**Table 4:** Calculated slope by Deming-regression for the tests with SPM and RPM in the knee rig. Additionally the results of the *t*-test against the slope of 1, which represents an identical behavior between both materials.

Parameter	Slope with standard deviation	*P* value
Quadriceps force	1.014 ± 0.086	0.69
Retropatellar peak pressure	0.977 ± 0.091	0.53
Contact area	1.051 ± 0.012	0.30
ap-translation	1.086 ± 0.207	0.31
Femorotibial rotation	0.956 ± 0.138	0.43
Shift of the patella	1.081 ± 0.169	0.25
Tilt of the patella	0.960 ± 0.176	0.57
Rotation of the patella	1.057 ± 0.243	0.57

**Table 5:** Load-dependent friction coefficient of UHMWPE against CoCr-alloy compared to references; the coefficients of friction were calculated from the determined exponential formulas of publications.

Pressure	Present study	Saikko [30]	Wang et al. [31]
3.1 MPa	0.099	0.158	0.083
4.7 MPa	0.093	0.119	0.063
6.2 MPa	0.080	0.099	0.052
7.8 MPa	0.073	0.084	0.044
9.4 MPa	0.065	0.074	0.039
